# iNKT and MAIT Cell Alterations in Diabetes

**DOI:** 10.3389/fimmu.2015.00341

**Published:** 2015-07-02

**Authors:** Isabelle Magalhaes, Badr Kiaf, Agnès Lehuen

**Affiliations:** ^1^INSERM U1016, Institut Cochin, Paris, France; ^2^UMR8104, CNRS, Paris, France; ^3^Laboratoire d’Excellence INFLAMEX, Université Paris Descartes, Sorbonne Paris Cité, Paris, France; ^4^Département de Diabétologie, Hôpital Cochin, Assistance Publique-Hôpitaux de Paris, Paris, France

**Keywords:** T1D, T2D, iNKT cells, MAIT cells, obesity, microbiota

## Abstract

Type 1 diabetes (T1D) and type 2 diabetes (T2D) are multifactorial diseases with different etiologies in which chronic inflammation takes place. Defects in invariant natural killer T (iNKT) cell populations have been reported in both T1D and T2D patients, mouse models and our recent study revealed mucosal-associated invariant T (MAIT) cell defects in T2D and obese patients. Regarding iNKT cells many studies in non-obese diabetic mice demonstrated their protective role against T1D whereas their potential role in human T1D is still under debate. Studies in mouse models and patients suggest that iNKT cells present in adipose tissue (AT) could exert a regulatory role against obesity and associated metabolic disorders, such as T2D. Scarce data are yet available on MAIT cells; however, we recently described MAIT cell abnormalities in the blood and ATs from obese and T2D patients. These data show that a link between MAIT cells and metabolic disorders pave the way for further investigations on MAIT cells in T1D and T2D in humans and mouse models. Furthermore, we hypothesize that the gut microbiota alterations associated with T1D and T2D could modulate iNKT and MAIT cell frequency and functions. The potential role of iNKT and MAIT cells in the regulation of metabolic pathways and their cross-talk with microbiota represent exciting new lines of research.

## Introduction

According to the WHO diabetes will be in 2030 the seventh leading cause of death (1). Type 1 diabetes (T1D) is a chronic autoimmune disease characterized by the destruction of the insulin-producing pancreatic β-cells, resulting in insulin deficiency and hyperglycemia. Type 2 diabetes (T2D) that accounts for 90–95% of all cases of diabetes, is characterized by insulin resistance, hyperglycemia, and decreased β-cell function and mass. The immune system is known to play a deleterious role in T1D as evidenced already in 1965 by Gepts who described insulitis in patients with T1D (2). More recent studies have shown that insulitis also occurs in patients with T2D, and support the notion that inflammation may participate in the pathogenesis of T2D (3, 4).

Invariant natural killer T (iNKT) and mucosal-associated invariant T (MAIT) cells are evolutionary conserved T cell subsets. iNKT and MAIT cells express semi-invariant T cell receptor (TCR) α chains: Vα24Jα18 and Vα7.2Jα33 in humans (Vα14Jα18 and Vα19Jα33 in mice), respectively (5, 6). Both display a memory phenotype, can readily produce cytokines, and thus, represent a bridge between innate and adaptive immunity. Based on mouse models, iNKT cells exert a regulatory role in T1D, while their role in T2D is still matter of debate. Regarding MAIT cells and diabetes, virtually nothing is known and the first insights regarding MAIT cells in T2D patients (7) have only recently been published.

Genetic and environmental factors are crucial in the development of T1D and T2D with growing evidence supporting an important role of the gut microbiota. This review will focus on iNKT and MAIT cells in the context of diabetes and discuss the potential impact of altered gut microbiota on these immune cells.

## iNKT Cell Defects in Non-Obese Diabetic Mice

The implication of iNKT cells in diabetes was first demonstrated in non-obese diabetic (NOD) mice that spontaneously develop T1D. Numerical and functional iNKT cell defects in NOD mice have been identified: reduced iNKT cell frequency and IL-4 production (8, 9). When compared with 37 other inbred mouse strains, in NOD mice iNKT cell numbers are at the low end of the spectrum in different tissues (i.e., peripheral blood, spleen, and thymus) (10). In NOD mice, defects in the expression of SLAM by double positive thymocytes that are responsible for the positive selection of NKT cells (11) and by myeloid dendritic cells (DCs) (12) are sought to play a role in the reduced iNKT cell number and impaired iNKT cell IL-4 production, respectively. Several other loci modulating iNKT cells in NOD mice have been identified (13, 14). More recently, Tsaih and colleagues demonstrated that a locus in chromosome 13 inversely regulates CD1d expression on double positive thymocytes and iNKT cell frequency, with the NOD allele shown to promote high CD1d expression on thymocytes and subsequent low iNKT cell frequency (15).

Of note, NOD mice have elevated frequency and number of an iNKT cell subpopulation producing IL-17, namely, iNKT17 cells, in the thymus and periphery (16). In the pancreatic lymph nodes, iNKT17 cells represent 13% of total iNKT cells in NOD mice as compared to 2% in C57BL/6 mice (16, 17).

## Regulatory Role of iNKT Cells in T1D in Mice

The accelerated development of T1D in CD1d-deficient NOD mice (18, 19), and the prevention of T1D development in NOD mice with increased iNKT cell number (20, 21) have suggested that iNKT cells play overall a protective role in T1D. T1D protection mediated by iNKT cells after cell transfer, upon cyclophosphamide treatment, or activation by α-galactosylceramide (α-GalCer), was shown to rely on IL-4 and/or IL-10 production (20, 22) and inhibition of pathogenic autoimmune responses (23, 24). Repetitive stimulation with α-GalCer-induced tolerogenic myeloid DCs (25) and plasmacytoid DCs that in turn converted naive BDC2.5 diabetogenic T cells into regulatory T (Treg) cells in pancreatic lymph nodes (26). Our group also demonstrated that iNKT cells could induce BDC2.5 T cell anergy in a cytokine-independent (i.e., IL-4, IL-10, IL-13, and TGF-β) (27) fashion, but required cell–cell contact and was independent of CD1d expression in the periphery, suggesting that molecular interactions other than CD1d/TCR are involved (28).

Environmental factors, such as viral infections, can be either deleterious or protective in T1D. Upon lymphochoriomeningitis virus infection, in pancreatic lymph nodes iNKT cells-induced tolerogenic plasmacytoid DCs, which converted naive T cells into Treg cells that migrated to the pancreatic islets and inhibited anti-islet T cells, thereby providing protection against T1D (29). Of note, even though a single injection of α-GalCer at the time of infection increased the frequency of Treg cells in pancreatic islets, and further promoted the protection against T1D, such protection was even seen in the absence of α-GalCer injection in wild type mice, but not in CD1d and Jα18 deficient NOD mice. Thus, iNKT cells are key in the induction of Treg cells and the protection against T1D in this infectious setting. We have also analyzed the role of iNKT cells upon another viral infection that is relevant to the human disease. Coxsackievirus B4 has been proposed as an etiologic agent that could promote the development of T1D in patients as well as in the diabetes susceptible NOD mouse. Coxsackievirus B4 infection accelerated T1D in NOD mice, whereas α-GalCer injection at the time of infection activated pancreatic iNKT cells that produced rapidly large amount of IFN-γ and upregulated indoleamine 2,3-dioxygenase production by macrophages recruited in the pancreas. These suppressive macrophages inhibited pancreatic anti-islet T cells and subsequently prevented T1D development (30). These data together showed that in both viral infections, through two different mechanisms, iNKT cells exert an efficient regulatory role.

However, not all iNKT cell subsets are protective. We showed that iNKT17 cells infiltrate the pancreas of NOD mice and promote diabetes development. α-GalCer treatment suppresses IL-17 (and to a lesser extend IFN-γ) produced by iNKT cells, which could also contribute to the protective role of α-GalCer in T1D (16). Of note, the presence of IL-1 and IL-6 in inflamed pancreatic islets of NOD mice may contribute to the activation of iNKT17 cells (31).

## Putative Role of iNKT Cells in Human T1D

The first data obtained in patients with T1D showed a decreased frequency of iNKT cells as well as a defect in IL-4 production (32) but since, contradictory results from clinical studies have been published; some following reports have supported this finding (33, 34), while one report has shown increased numbers of iNKT cells (35), and others did not find differences in iNKT cell numbers (36–39).

While the frequency of iNKT17 cells might be extremely low in the peripheral blood of healthy controls and patients with T1D, iNKT17 cells could be expanded *in vitro* in the presence of IL-1β. These cells were only obtained from the blood of T1D patients but not from healthy controls (40), suggesting that iNKT17 cells could also be involved in T1D pathogenesis in patients.

Altogether, despite converging evidence that iNKT cells play a regulatory role in T1D using mouse models, their role in human T1D remains controversial urging more clinical studies with well defined T1D patient cohorts.

## iNKT Cells in T2D and Obesity

Type 2 diabetes is a progressive disease resulting from the insulin resistance that develops with advancing age and lifestyle factors, such as inactivity, diet, and obesity (most patients with T2D are obese or overweight), but those factors are not the only trigger. It is now recognized that T2D results from the interaction between different genetic events and with environmental factors (41). The detection of TNF-α in obese rat adipose tissue (AT) provided the first evidence that tissue inflammation was correlated with insulin resistance and T2D (42). In the lean state M2 macrophages with an anti-inflammatory phenotype accumulate in AT, whereas obesity leads to the preferential accumulation in AT of proinflammatory M1 macrophages known to participate in insulin resistance development. Other immune cells infiltrate AT, and iNKT cells are particularly enriched in white AT.

In obese mice, iNKT cell frequency in white AT is decreased while weight loss reverses decreased AT iNKT cell frequency (43). Several studies have analyzed the impact of iNKT cells in metabolic control with contradictory results. The use of CD1d^−/−^ or Jα18^−/−^ mice lacking all NKT cells (iNKT and variant NKT cells) or only iNKT cells, respectively, and other factors, such as different diets, or experimental procedures have been implicated to explain the protective, the absence, or the negative impact of iNKT cells on weight gain or metabolic control (44, 45). In a recent review, Lynch argues that despite the divergent results obtained using iNKT-deficient mouse models, most experiments using transferred or activated α-GalCer iNKT cells converge to support a protective role of iNKT cells in obesity and she proposes that AT iNKT cells via IL-4 and IL-10 production regulate anti-inflammatory cytokines and adipocyte function (46). The regulatory role of AT iNKT cells is supported by recent findings showing that in murine AT, iNKT cells did not express the PLZF transcription factor, characteristic of iNKT cells, but instead the transcription factor E4BP4, and via IL-10 and IL-2 expression control the homeostasis of macrophages and Treg cells, respectively (47).

In obese patients as compared to lean individuals, iNKT cell frequency is decreased in omental AT and peripheral blood (7, 48). Conversely, iNKT cell frequency in peripheral blood is restored after bariatric surgery of obese patients (43).

## MAIT Cells in T1D

Due to the lack of specific antibodies directed against the murine Vα19 TCR chain, limited data on murine MAIT cells are available. However, the recent development of mouse MR1-antigen loaded tetramers detecting specifically MAIT cells (49) will most likely soon shed a new light on the role of MAIT cells in different mouse disease models, such as diabetes.

To date, only scarce data on the role of MAIT/MAIT-like cells in T1D are available. The observation that the expression of Vα19Jα33 TCR as a transgene in NOD mice delays the onset of T1D (50) suggests that MAIT cells may play a protective role. In humans, MAIT cells are identified using anti-Vα7.2 TCR chain and anti-CD161 antibodies. A recent report analyzed that the CD161^bright^CD8^+^ T cell subset in juvenile T1D patients (51), with the CD161^bright^CD8^+^ T cells displaying a phenotype, IL-18Rα^+^, CD127^+^, CD45RA^−^, and CCR7^−^, suggestive of MAIT cells. No difference in the CD161^bright^CD8^+^ T cell frequency was observed in juvenile T1D patients as compared to age-matched controls. As described previously for MAIT cells (52), the frequency of CD161^bright^CD8^+^ T cells increased with age in juvenile controls and new-onset T1D patients but not in juvenile long-standing (≥1 year) T1D patients. These results suggest that in long-standing T1D patients the circulating CD161^bright^CD8^+^ T cells may be depleted. The CD27^−^ CD161^bright^CD8^+^ T cells (a subset enriched in IL-17 producing cells) were increased in patients with T1D as compared to controls. Further studies of T1D patients using anti-Vα7.2 TCR chain and anti-CD161 antibodies and/or with human MR1-antigen loaded tetramers specifically directed toward MAIT cells are needed in order to accurately decipher their role in T1D.

Additionally, whether MAIT cells as seen for IL-17-producing γδ T cells exit the thymus as CD27^−^ cells (53), or acquire the CD27^−^ phenotype in the periphery upon activation and differentiation as observed for Th17 cells (54) and the understanding of the underlying mechanisms would be of utter interest.

## MAIT Cell Defects in T2D and Obese Patients

Our group has shown that MAIT cells exhibit several defects in T2D and obese patients (7). MAIT cell frequency was dramatically reduced in patients with T2D, and particularly in obese patients. In 12/69 severe obese patients study, we could not detect circulating MAIT cells. Higher frequencies of MAIT cells producing IL-17 were detected in T2D and obese patients, as compared to lean control individuals, and this was even more pronounced in T2D patients. Furthermore, when stimulated *in vitro* with MAIT cell ligand, a higher frequency of T2D patient MAIT cells produced IL-17. We showed that MAIT cells are present in the omental and subcutaneous AT, with comparable frequencies between lean control individuals and obese patients. Interestingly, in five obese patients for whom we could not detect circulating MAIT cells, MAIT cells were present in omental AT. In AT, and particularly subcutaneous AT, a vast majority of MAIT cells from obese patients, but not from lean control individuals, produced IL-17. AT from obese patients promoted MAIT cell activation (upregulation of CD25 and CD69 expression), and as compared to circulating MAIT cells, AT MAIT cells displayed higher Ki67 expression, altogether suggesting a recruitment, and local activation of MAIT cells in the AT. Bariatric surgery of obese patients restored circulating MAIT cell frequency and decreased their production of IL-2 and granzyme B. However, up to 12 months post-surgery high frequencies of MAIT cells still displayed an augmented Th17 profile. A recent report by Carolan et al. confirms our observation on MAIT cell alteration in adult obesity, showing decreased MAIT cell frequency and increased IL-17 production (55). However, the frequency of circulating MAIT cells in obese children was increased as compared to lean children, and this increased frequency in patients was associated with hyperinsulinemia and insulin resistance. Their analysis of adult obese patients’ AT confirms the increased production of IL-17 by MAIT cells and shows a decreased frequency of IL-10 producing MAIT cells.

The mechanisms underlying MAIT cells defects and increased IL-17 production in T2D and obesity remain to elucidate. For instance, whether the expression of IL-1β (56) and IL-6 (57) in the AT of T2D patients plays a role in MAIT cell activation and production would be of interest. Altogether, these data show alterations of the MAIT cell compartment in T2D and obese patients and paves the way for further studies assessing the role of MAIT cells in diabetes and metabolic disorders.

## iNKT and MAIT Cells and Impact of Microbiota in T1D and T2D

Type 1 diabetes and T2D are linked to genetic predisposition but non-genetically determined factors, such as the gut microbiota, also impact their development. We will discuss below how altered gut microbiota may impact iNKT cells and MAIT cells in T1D and T2D.

Invariant natural killer T and MAIT cells are present in human and murine AT and intestine. In mice, microbial exposure early in life impacts iNKT cell numbers (58), and in germ-free mice MAIT cells are absent in peripheral tissues (59). Altogether, these results show that commensal bacteria impact intestinal iNKT cell homeostasis, and are essential for MAIT cell expansion. The microbial lipids activating intestinal iNKT cells remain to be elucidated, but recent reports have shown that, in a CD1d-dependent fashion, some sphingolipids from the gut commensal *Bacteroides fragilis* activate (60) while other sphingolipids inhibit iNKT cells (61). MAIT cell ligands are MR1-restricted derived bacterial products of vitamin B metabolism (62). The 6-formyl pterin, a folic acid (vitamin B9) metabolite is a non-activating ligand, while ligands derived from the riboflavin (vitamin B2) synthesis pathway, such as ribityllumazines and pyrimidines, activate MAIT cells. Pyrimidines that represent the most potent MAIT cell activating ligands are formed from the condensation of an intermediate of riboflavin synthesis (5-amino-6-d-ribitylaminouracil) with the glucose-derived methylglyoxal or glyoxal (63).

Alterations in the gut microbiota of NOD mice have been shown to be strongly associated with the development of T1D. This was supported by gut microbiota transfer experiments from T1D-protected animals into young diabetes-prone mice that upon transfer showed delayed T1D or protection from T1D (64, 65). The impact of the human intestinal microbiota on different diseases including T1D development is a field of intensive investigation and cohort studies designed to address its role on T1D are currently underway (66). Several studies analyzing the fecal bacteria composition and metagenomic support the association between changes in intestinal microbiota and risk of T1D (67, 68). Similarly, alterations of the gut microbiota composition have been reported in obese mice, obese patients, and patients with T2D (69). In turn, those alterations in microbiota may impact gut permeability in T1D, obesity, and T2D; increased bacterial translocation is thought to contribute to the establishment of AT microbiota (70). Two metagenome studies of T2D patients and healthy individuals have revealed differences in microbial functions related to vitamin metabolism (including riboflavin) (71, 72). Interestingly, in T1D and T2D patients the production and plasma levels of methylglyoxal are elevated (73).

**Figure 1:**
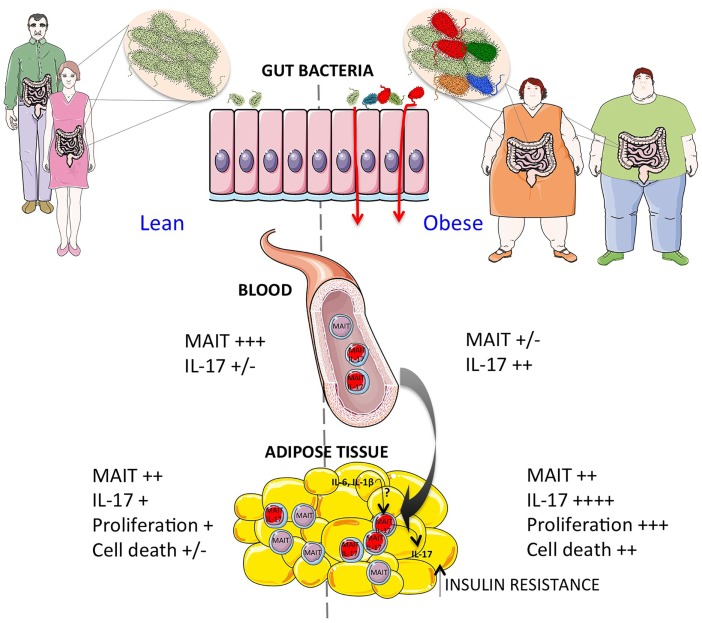
**Increased activation of MAIT cells in obesity**. Obese patients show a dramatic decrease of circulating MAIT cells correlated with increased IL-17 production. The modifications of gut microbiota may contribute to impaired gut integrity and increased MAIT cell activation. In the adipose tissue of obese patients, the exacerbated MAIT cell IL-17 production may contribute to the insulin resistance. Moreover, the decreased Bcl-2 expression, and increased Ki67 expression in adipose tissue as compared to blood suggest that locally MAIT cells may be activated and undergo cell death by mechanisms yet to be determined.

The gut microbiota plays a crucial role on iNKT and MAIT cell development, and iNKT and MAIT cells activating and inhibiting microbial-derived antigens have been identified. Therefore, it is tempting to speculate that in T1D and T2D alterations of the gut microbiota, and possibly also AT associated microbiota, impact iNKT and MAIT cells homeostasis in the gut and AT. We hypothesize that in diabetes and obesity MAIT cells are recruited to the gut and/or AT therefore depleting the circulating compartments, and in the AT of obese patients the exacerbated IL-17 production by MAIT cells participate to the local inflammation and insulin resistance (Figure 1).

Invariant natural killer T cells have been shown to influence the gut microbial colonization (74). It would be very interesting to assess if MAIT cells can also modulate the gut microbiota. A recent report showed that circulating MAIT cell deficiency observed in patients with systemic lupus erythematosus (SLE) and patients with rheumatoid arthritis (RA) was associated with circulating iNKT cell deficiency in patients with SLE but not in patients with RA (75). Furthermore, the authors showed that iNKT cell activation by α-GalCer induces MAIT cell activation. In obese patients, we did not find a correlation between the decreased iNKT and MAIT cell frequency (unpublished data).

Gut microbiota dysbiose and defective gut integrity observed in T1D and T2D could lead to the presence of TLR ligands in the blood and peripheral tissues, thereby inducing iNKT and MAIT cell activation indirectly through DC. However, it is interesting to note that in our recent study in obese and T2D patients, the alteration observed in iNKT and MAIT cells was not observed in other T cell populations, such as conventional T cells and γδT cells. This data suggest that specific iNKT and MAIT cell ligands could play a key role in their alteration.

Invariant natural killer T and MAIT cells are evolutionary conserved populations of innate-like T cells supporting an important role as a first line of defense against pathogens. The abundance of iNKT and MAIT cells not only in the AT but also in the liver prompts the question if iNKT and MAIT cells may engage in a cross-talk and/or in the regulation of metabolic pathways.

## Conflict of Interest Statement

The authors declare that the research was conducted in the absence of any commercial or financial relationships that could be construed as potential conflict of interest.
